# Successful vitrification of pronuclear-stage pig embryos with a novel cryoprotective agent, carboxylated ε-poly-L-lysine

**DOI:** 10.1371/journal.pone.0176711

**Published:** 2017-04-27

**Authors:** Maki Kamoshita, Tsubasa Kato, Katsuyoshi Fujiwara, Takafumi Namiki, Kazuaki Matsumura, Suong-Hyu Hyon, Junya Ito, Naomi Kashiwazaki

**Affiliations:** 1 Laboratory of Animal Reproduction, Graduate School of Veterinary Science, Azabu University, Sagamihara, Japan; 2 Japan Advanced Institute of Science and Technology, Nomi, Japan; 3 Center for Fiber and Textile Science, Kyoto Institute of Technology, Matsugasaki, Kyoto, Japan; 4 School of Veterinary Medicine, Azabu University, Sagamihara, Japan; Peking University Third Hospital, CHINA

## Abstract

Vitrification is a powerful tool for the efficient production of offspring derived from cryopreserved oocytes or embryos in mammalian species including domestic animals. Genome editing technologies such as transcription activator-like effector nucleases (TALENs) and clustered regularly interspaced short palindromic repeats (CRISPR)/ CRISPR-associated (Cas)9 are now available even for domestic species, suggesting that the vitrification of embryos at the pronuclear stage (PN) will be more important because they could provide genomic host cells to be targeted by TALENs or CRISPR/Cas9. Although we reported the successful production of piglets derived from vitrified PN embryos by a solid-surface vitrification method with glutathione supplementation, further improvements are required. The cryoprotective agent (CPA) carboxylated ε-poly-L-lysine (COOH-PLL) was introduced in 2009. COOH-PLL reduces the physical and physiological damage caused by cryopreservation in mammalian stem cells and the vitrification of mouse oocytes and embryos. Those results suggested that vitrification of COOH-PLL may help improve the developmental ability of pig embryos vitrified at the PN stage. However, it remains unclear whether COOH-PLL is available as a CPA for the vitrification of embryos in domestic species. In this study, we evaluated COOH-PLL as a CPA with ethylene glycol (EG) and Cryotop as a device for the vitrification of PN pig embryos. Exposure to vitrification solution supplemented with COOH-PLL up to 30% did not decrease developmental ability to the 2-cell stage and the blastocyst stage. After warming, most of the vitrified embryos survived regardless of the concentration of COOH-PLL (76.0 ± 11.8% to 91.8 ± 4.6%). However, the vitrified embryos without COOH-PLL showed a lower development rate up to the blastocyst stage (1.3 ± 1.0%) compared to the fresh embryos (28.4 ± 5.0%) (*p*<0.05). In contrast, supplementation of 20% (w/v) COOH-PLL in the vitrification solution dramatically improved the developmental ability to blastocysts of the vitrified embryos (19.4 ± 4.6%) compared to those without COOH-PLL (*p*<0.05). After the transfer of embryos vitrified with 30% (v/v) EG and 20% (w/v) COOH-PLL, we successfully obtained 15 piglets from 8 recipients. Taken together, our present findings demonstrate for the first time that COOH-PLL is an effective CPA for embryo vitrification in the pig. COOH-PLL is a promising CPA for further improvements in the vitrification of oocytes and embryos in mammalian species.

## Introduction

For the production of transgenic animals, pre-implantation embryos at the pronuclear (PN) stage are usually used, as the injection of foreign DNA into pronuclei contributes to the recombination of genomic DNA [1]. It was demonstrated that PN embryos can be used not only for transgenic animals but also for knockout animals by using a genome-editing system such as zinc-finger nucleases (ZFNs) [2], transcription activator-like effector nucleases (TALENs) [3], or clustered regularly interspaced short palindromic repeats (CRISPR)/ CRISPR-associated (Cas)9 [4] through the induction of these nucleotides. Even in the pig, gene-modified pigs have been successfully produced using a genome-editing system via microinjection or somatic cell nuclear transfer [5–8] because gene-modified pigs can contribute to the expansion of lots of biomedical researches, for example generating human organs and tissues, designing new dung screening methodologies and developing new human disease models [9]. In addition, it has been recently reported successful interspecies human-pig blastocyst complementation [10]. These results lead us the idea that the cryopreservation of embryos will become more important, because such embryos are ready-to-use after warming. However, it is well known that pig embryos are very sensitive to damage caused by low temperature and osmotic stress [11].

The vitrification method is now common for the cryopreservation of oocytes and embryos instead of conventional freezing methods. The vitrification method was first reported by Rall and Fahy [12]. The major advantages of the vitrification method are (1) the elimination of the physiological damage caused by intracellular or extracellular ice crystal formation, and (2) the reduction of chilling damage by shortening the exposure to suboptimal temperature [13]. One of the major factors that can affect the efficiency of vitrification is the cryoprotective agent (CPA). Various CPAs such as dimethyl sulfoxide (DMSO), ethylene glycol (EG), glycerol, and propylene glycol have been widely used for the vitrification of oocytes and embryos in various mammalian species [14–16].

Our previous study demonstrated that compared to DMSO, EG had a lesser toxic effect on the vitrification of unfertilized oocytes in mice [17]. It was also suggested that the current CPAs, even EG, have toxic effects on cell viability in a dose-dependent manner [18], indicating that the development of a new CPA showing high efficiency and low toxicity is necessary for further improvements in vitrification. Carboxylated ε-poly-L-lysine (COOH-PLL) was introduced as a CPA in 2009 by Matsumura and Hyon [18]. They demonstrated that COOH-PLL reduced the risks of damage by ice recrystallization during freezing and thawing, with anti-freezing protein-like activities [19]. We succeeded in producing mouse offspring derived from unfertilized oocytes and PN embryos vitrified with COOH-PLL and EG [20, 21]. Although the production of piglets derived from vitrified PN embryos was reported using another vitrification method and CPA, i.e., a SSV method and EG [22], our results from the mouse indicated that COOH-PLL is a more suitable CPA for the vitrification of oocytes and embryos, even in the pig, compared to the other CPAs that have been used to date. We speculated that vitrification using COOH-PLL as a CPA could thus provide a high success rate for embryo vitrification even in pigs.

The objective of the present study was to clarify whether COOH-PLL is effective as a CPA for the vitrification of PN porcine embryos. We also evaluated the *in vitro* and *in vivo* development of PN porcine embryos that were vitrified using COOH-PLL.

## Materials and methods

All chemicals and reagents were purchased from Sigma-Aldrich (St. Louis, MO, USA) unless otherwise stated. The study was approved by the Ethical Committee for Vertebrate Experiments at Azabu University (ID#140219–4) [23].

### Oocyte collection and *in vitro* maturation (IVM)

The collection of porcine follicular oocytes and *in vitro* maturation (IVM) were performed as described by Kikuchi *et al* [24]. In brief, porcine ovaries were collected at a local slaughterhouse and transported to the laboratory at 37.5°C. Cumulus oocyte complexes (COCs) were collected from 2–6 mm in diameter follicles. Fifty COCs were cultured for 22 h in four-well dishes (Nunc™ Cell-Culture Treated Multidishes; Thermo Fisher Scientific Inc., Waltham, MA, USA), each containing 500 μL of a modified North Carolina State University-37 (NCSU-37) solution [25] which contained 10% (v/v) porcine follicular fluid, 0.6 mM cysteine, 20 μM beta-mercaptoethanol, 1 mM dibutyryl cAMP (dbcAMP), 10 IU/mL eCG (1000 units; PMS; Nippon Zenyaku Kogyo, Fukushima, Japan), and 10 IU/mL hCG (3000 units; Puberogen; Novartis Animal Health, Tokyo). The COCs were subsequently cultured for 22 h in NCSU-37 solution without dbcAMP, eCG or hCG. The maturation culture was performed under 5% CO_2_ in air at 38.5°C.

### *In vitro* fertilization and *in vitro* culture

The *in vitro* fertilization (IVF) and *in vitro* culture (IVC) were performed as described by Kikuchi *et al* [24]. After 44 h IVM, COCs were washed three times in modified pig fertilization medium (Pig-FM) [26] and 20–25 COCs were transferred into each 90 μL droplet of Pig-FM covered with paraffin oil (Kanto Chemicals, Tokyo). Epididymal spermatozoa were collected and frozen as described by Kikuchi *et al* [27]. Frozen-thawed epididymal spermatozoa were washed in Medium 199 with Earle salts (Gibco) adjusted to pH 7.8 [22] and preincubated for 15 min at 38.5°C in Pig-FM.

After preincubation, 10 μL of sperm was added to the droplets of Pig-FM containing COCs. The final concentration of sperm was 1.0 x 10^6^ sperm/mL. The COCs and sperm were co-cultured for 3 h at 38.5°C under 5% CO_2_ in air. At 3 h after the IVF, the cumulus cells and sperm were removed from the oocytes with the use of a fine glass pipette. Denuded oocytes were cultured in NCSU-37 without glucose supplemented with 50 μM beta-mercaptoethanol, 0.17 mM sodium pyruvate, 2.73 mM sodium lactate, and 4 mg/mL albumin from bovine serum (BSA) (IVC-PyrLac) [24] for 48 h.

The embryos were subsequently cultured in NCSU-37 supplemented with 5.55 mM glucose, 50 μM beta-mercaptoethanol and 4 mg/mL BSA (IVC-Glu) [24] for 120 h. IVC was performed at 38.5°C under 5% CO_2_ in air. At 10 h after the IVF, the oocytes were centrifuged at 17,860 *g* at 38°C for 10 min in a 1.5-mL tube for the visualization of the pronuclei. Oocytes with two or three pronuclei (PN) were defined as porcine embryos at the PN stage and further used for vitrification [22].

### Vitrification and warming

Vitrification and warming by the Cryotop method was performed as describe [28] with some modification. PN embryos were selected and exposed for 10 min to PB1, i.e., phosphate-buffered saline (PBS) supplemented with 20% (v/v) fetal calf serum (FCS), 15% (v/v) ethylene glycol (EG) and COOH-PLL. Embryos were exposed to PB1 supplemented with 20% (v/v) FCS, 0.5 M sucrose, 30% (v/v) EG and COOH-PLL for 1 min before being plunged into liquid nitrogen on Cryotop. In each Cryotop, 10–20 embryos were loaded.

The concentrations of COOH-PLL in the vitrification solution (w/v, P0, P1, P10, P20 and P30, respectively), and the concentrations in the equilibration solution were one-half of those in the vitrification solution (Table 1). Vitrified embryos were preserved in liquid nitrogen for at least 1 week. Embryos that had been vitrified on the Cryotop were immersed in PB1 supplemented with 20% (v/v) FCS and 1.0 M sucrose for 1 min. The embryos were then transferred to PB1 supplemented with 20% (v/v) FCS and 0.5 M sucrose for 3 min, and transferred to supplemented PB1 with 20% (v/v) FCS for 5 min. After warming, the embryos were cultured at 38.5°C under 5% CO_2_ in air. To investigate the effect of COOH-PLL on the developmental ability of the embryos, some embryos were only exposed to equilibration solution, and not vitrified.

**Table 1:** The concentrations of the cryoprotectants in the equilibration solution (ES) or vitrification (VS).

Treatments	ES^1^	VS^2^
P0	15%EG	30%EG
P1	15%EG + 0.5%COOH-PLL	30%EG + 1%COOH-PLL
P10	15%EG + 5%COOH-PLL	30%EG + 10%COOH-PLL
P20	15%EG + 10%COOH-PLL	30%EG + 20%COOH-PLL
P30	15%EG + 15%COOH-PLL	30%EG + 30%COOH-PLL

^1^These cryoprotectants were added into PB1 supplemented with 20% FCS.

^2^These cryoprotectants were added into PB1 supplemented with 20% FCS and 0.5 M sucrose.

### Evaluation of survival and developmental ability *in vitro*

We morphologically evaluated the survival of the vitrified embryos at 1 h after warming.

The membrane integrity of vitrified-warmed embryos under a microscope according to the method by Zeron *et al* [29]. Embryos with normal and spherical shape, without lysis, and not shrunken, swollen, or blackened were regarded as surviving. To evaluate the developmental ability of vitrified or exposed embryos *in vitro*, we determined the cleavage rates (at 38 h after warming) and the rates of blastocyst formation (at 158 h after warming). Blastocysts were harvested and stained with orcein for the determination of the number of cells in each blastocyst [22].

### Evaluation of the permeability of COOH-PLL to embryos

COOH-PLL was prepared as reported previously [20]. To clarify the permeability of PN embryos to COOH-PLL as a CPA, we used fluorescein isothiocyanate (FITC)-labeled COOH-PLL. After centrifugation, PN embryos were exposed to PB1 supplemented with FITC-labeled COOH-PLL (5% (w/v)) for 5 min. After exposure, some of the embryos were washed 3 times with PBS. Both washed and unwashed embryos were used for the experiments to evaluate the interaction of COOH-PLL with the embryos under confocal laser microscopy (TCS-SP5; Leica Co. Ltd, Wetzlar, Germany). As control, mouse PN embryos were also collected by in vitro fertilization as previously reported [20] and then used for the experiments as described above.

### Embryo transfer

The embryo transfer was performed as described [30]. Estrus synchronization of the recipient nonpregnant gilts (>140 days old) was achieved by an intramuscular injection of 1,500 IU eCG followed by an injection of 500 IU hCG 72 h later. Embryos that were vitrified with P20 and warmed were surgically transferred into the oviducts of estrus-synchronized recipient gilts. After warming, 97–143 embryos (5–10 Cryotops) were transferred within 1h for each experiment. On Day 28 (Day 0 was the day of transfer), pregnancy was confirmed in the recipients with an ultrasound pregnancy detector (Doppler Pregtector; Rotech Livestock Equipment, Chichester, England). The offspring was confirmed on Days 115–116.

### Statistical analysis

Each experiment had at least five replicates. All percentage data were subjected to arcsine transformation before the statistical analysis. Kruskal-Wallis test or Scheffe’s method was used for the analysis. Data are shown as mean ± standard error of the mean (SEM).

## Results

### The developmental ability of PN embryos exposed to various concentrations of COOH-PLL

**Fig 1:**
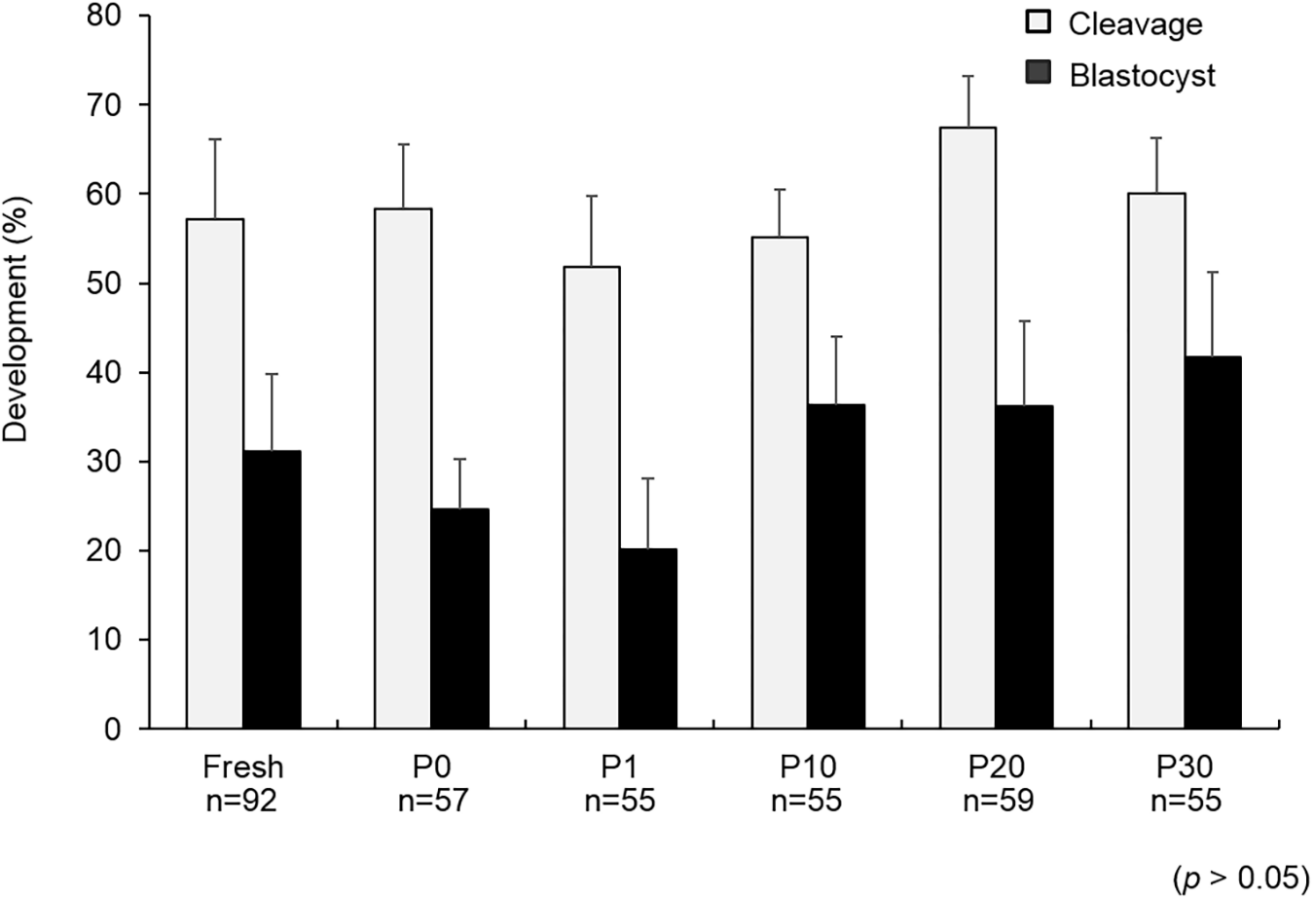
The developmental ability of PN porcine embryos exposed to various concentrations of COOH-PLL. Data are mean ± SEM. Data was analyzed by Kruskal-Wallis test. There were no significant differences in the cleavage rate or blastocyst rate among the treatments (*p*>0.05). Number of oocytes used in each group were described under each treatment group.

**Fig 2:**
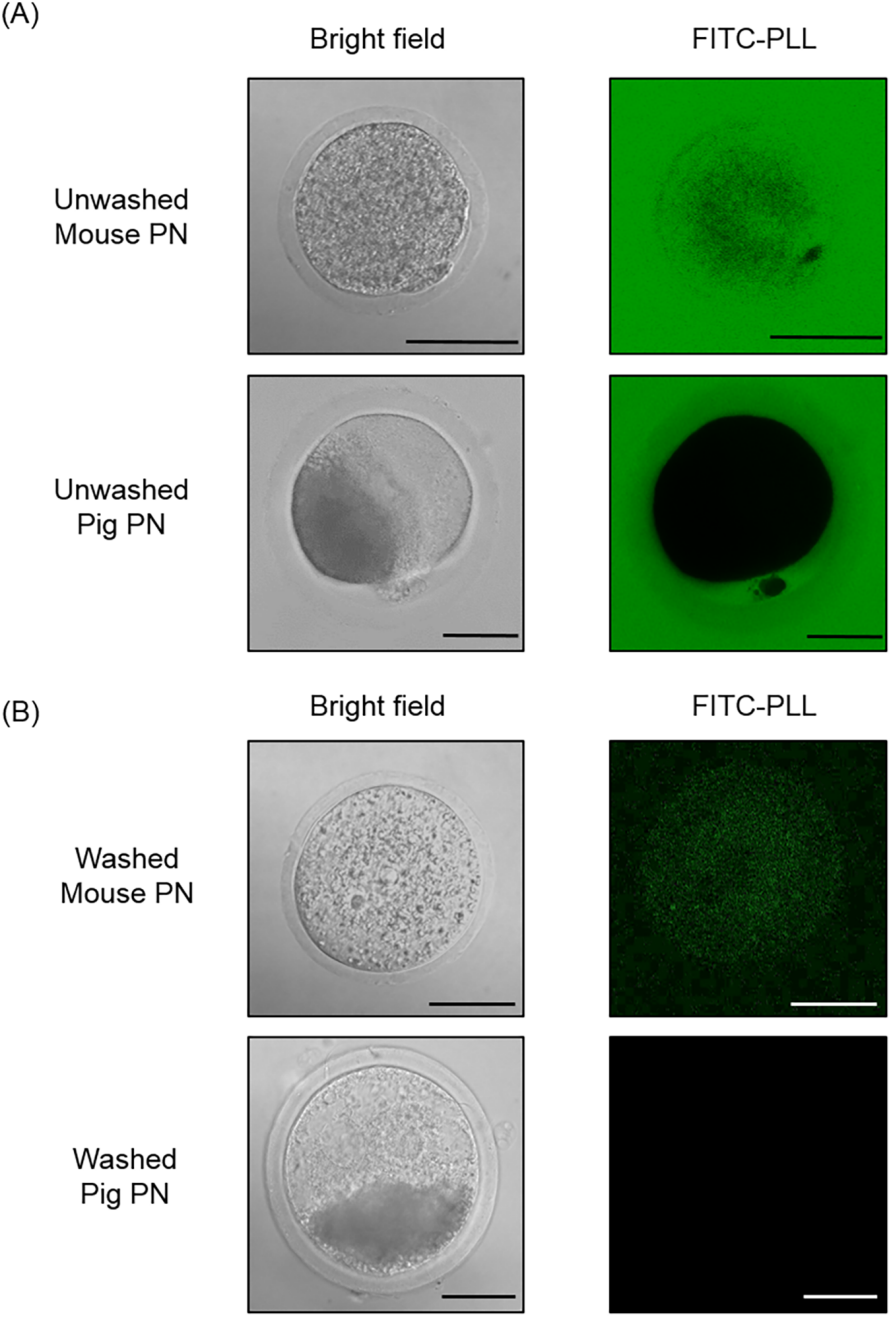
The permeability of PN mouse and pig embryos to COOH-PLL. The embryos were exposed to FITC-labeled COOH-PLL (5% (w/v)) for 5 min. After exposure, embryos with or without washing were examined under a laser scanning microscope. Scale bars denote 50 um.

There were no significant differences (*p*>0.05) in the cleavage rates of the PN embryos exposed to different concentrations of COOH-PLL (P0: 59.6 ± 7.1%, P1: 52.7 ± 5.3%, P10: 56.4 ± 5.8%, P20: 67.8 ± 5.8%, and P30: 60.0 ± 6.2%, respectively) (Fig 1). There were also no significant differences (*p*>0.05) in the blastocyst rates of the PN embryos exposed to different concentrations of COOH-PLL (P0: 24.6 ± 5.6%, P1: 20.0 ± 8.0%, P10: 36.4 ± 7.7%, P20: 37.3 ± 9.6%, and P30: 41.8 ± 9.5%, respectively) (Fig 1). To confirm whether COOH-PLL enters to the cytoplasm of embryos, we exposed mouse and pig PN embryos to FITC-tagged COOH-PLL. The results are illustrated in Fig 2. FITC-tagged COOH-PLL was partially observed in cytoplasm of both unwashed and washed mouse embryos (Fig 2A and 2B). On the other hand, FITC was not observed in cytoplasm of both unwashed and washed pig embryos.

### The survival and developmental ability *in vitro* of the PN embryos vitrified with various concentrations of COOH-PLL

**Fig 3:**
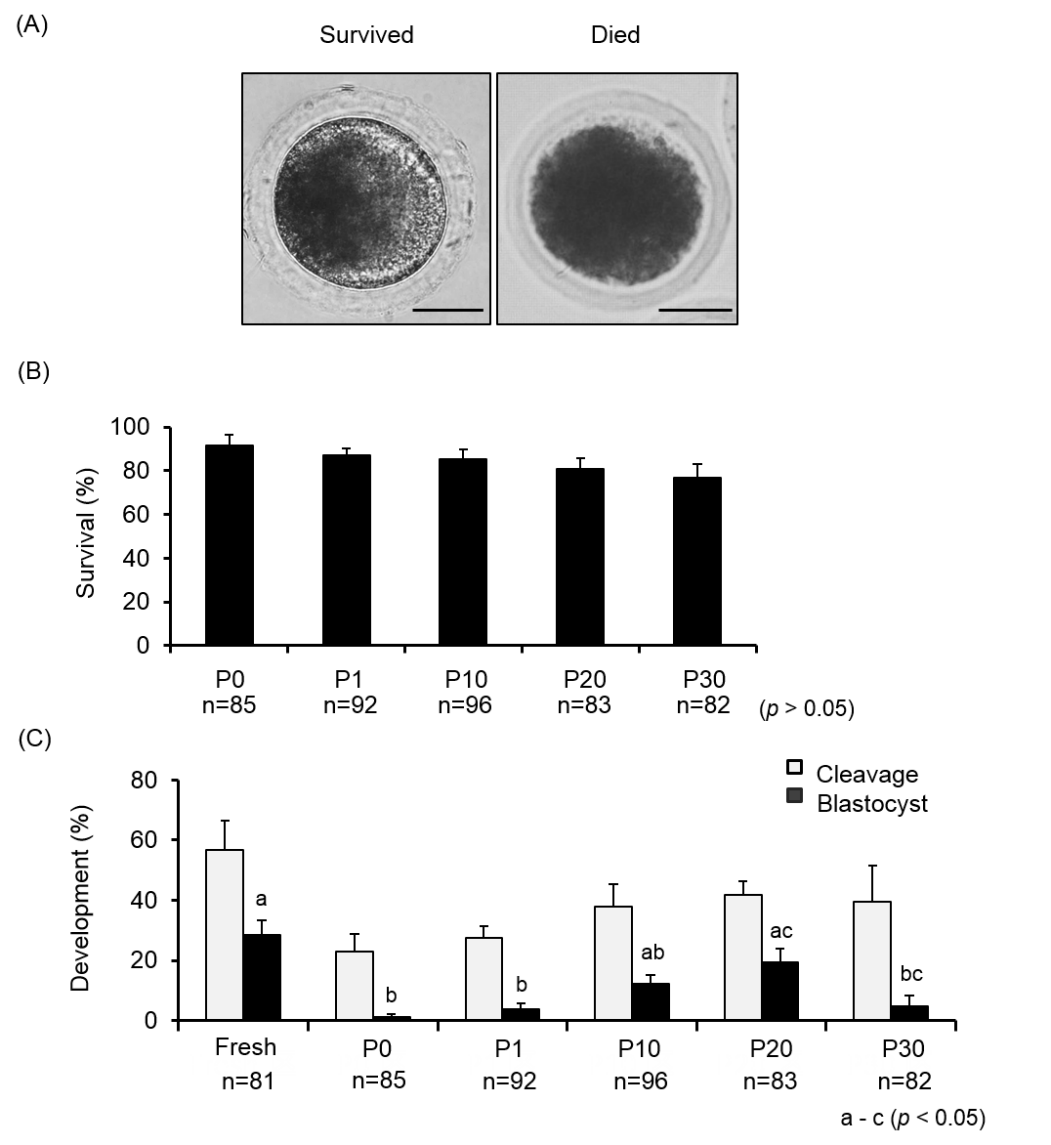
**The effects of COOH-PLL on the morphology (A), survival (B) and developmental abilities (C) of vitrified porcine embryos at the PN stage.** Data are mean ± SEM. Scheffe’s method was used for the analysis. Different superscripts denote significant differences (*p<*0.05). Number of oocytes used in each group were described under the treatment group. Scale bars denote 50 um.

Typical morphology of embryos after vitrification was shown in Fig 3A.

Embryos with normal and spherical shape, without lysis, and not shrunken, swollen, or blackened were regarded as surviving (Fig 3A) and others were defined as non-survived (Fig 3A).

There were no significant differences (*p*>0.05) in the survival rates of the PN embryos vitrified with different concentrations of COOH-PLL (P0: 91.8 ± 4.6%, P1: 91.7 ± 5.7%, P10: 83.2 ± 3.6%, P20: 72.7 ± 7.3%, and P30: 76.0 ± 11.8%, respectively) (Fig 3B). There were also no significant differences (*p*>0.05) in the cleavage rates of the PN embryos vitrified with different concentrations of COOH-PLL (P0: 23.1 ± 5.8%, P1: 27.5 ± 3.8%, P10: 37.8 ± 7.5%, P20: 41.8 ± 4.6% and P30: 39.7 ± 11.8%) (*p*>0.05) (Fig 3C).

The blastocyst rate of the PN embryos vitrified with P20 (19.4 ± 4.6%) was significantly higher than that of the P0 (1.3 ± 1.0%) and P1 (3.8 ± 2.1%) groups (*p<*0.05). There were no significant differences (*p*>0.05) in blastocyst rates between the P20 group and the fresh group (28.4 ± 5.0%) (Fig 2B). The total numbers of cells in the blastocysts developed from the embryos vitrified with P10 (22.7 ± 2.3 cells) were significantly lower than those of the fresh embryos (36.1 ± 2.9 cells) (*p<*0.05), but there was no difference in the total numbers of cells between the P20 group (30.2 ± 2.1 cells) and the fresh group (*p*>0.05) (Table 2).

**Table 2:** The effect of COOH-PLL concentration on the number of cells in a blastocyst.

	Blastocysts	No. of total cells
Fresh	31	36.1 ± 2.9^a^
P10	14	22.7 ± 2.3^b^
P20	24	30.2 ± 2.1^ab^
P30	13	21.8 ± 2.2^b^

Data are shown as mea ns ± S.E.M. Different superscripts denote a significant difference (*p<*0.01) by Scheffe’s method.

### The *in vivo* developmental ability of PN embryos vitrified with COOH-PLL

**Fig 4:**
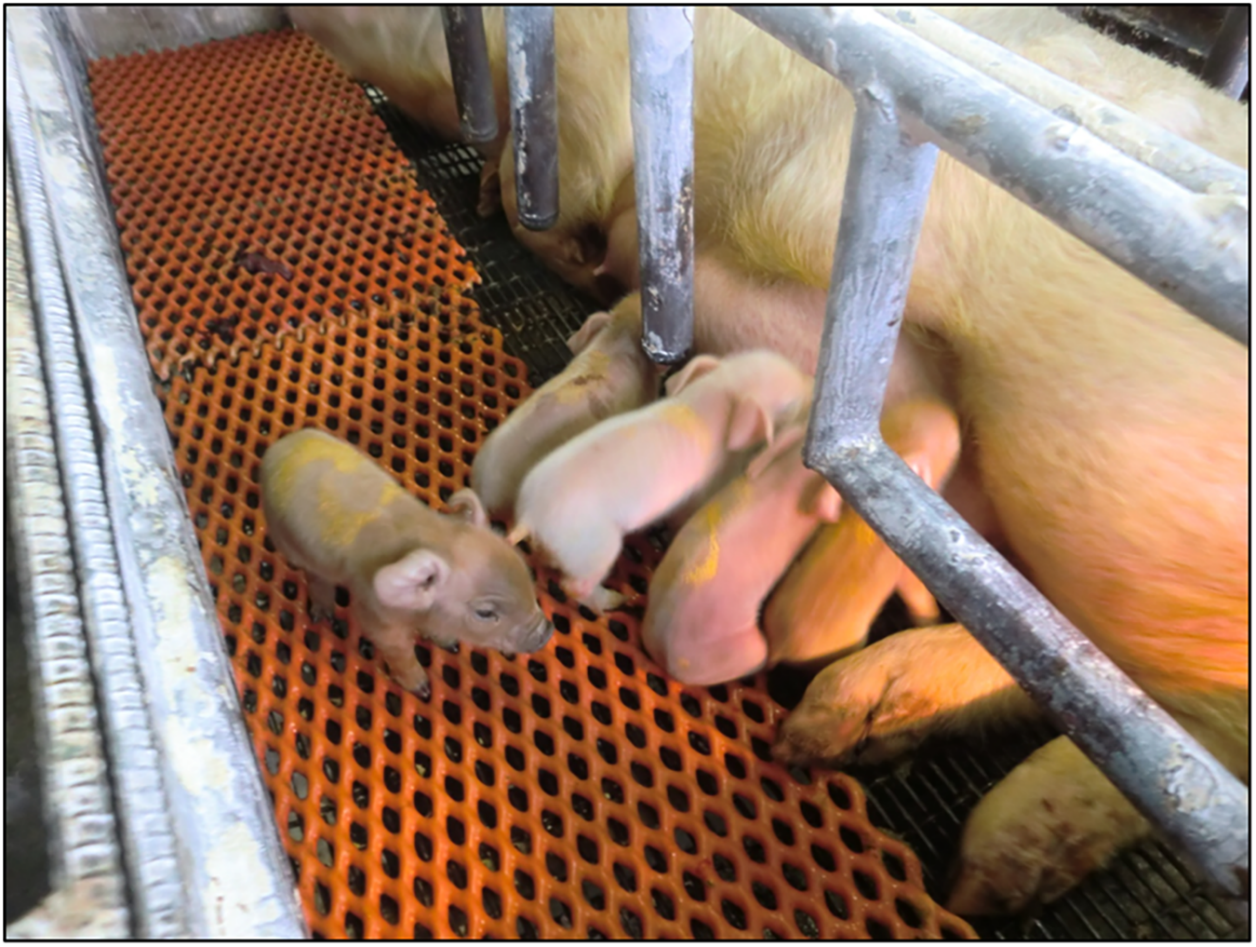
Piglets derived from PN porcine embryos vitrified with COOH-PLL.

In our preliminary study, 79 fresh embryos were transferred to a recipient and 13 piglets were obtained (data not shown). The transfer of PN embryos vitrified with P20 into eight recipients resulted in two pregnancies, which were maintained until term (Table 3). Fifteen piglets were obtained from the two pregnant recipients; two of them were stillborn (Fig 4).

**Table 3:** *In vivo* development of porcine embryos vitrified with P20.

Recipients	No. of embryos transferred	Pregnant (Yes/No)	No. of piglets (No. of stillborn)
#1	143	No	0 (0)
#2	103	No	0 (0)
#3	111	No	0 (0)
#4	105	Yes	5 (0)
#5	121	No	0 (0)
#6	121	No	0 (0)
#7	117	Yes	10 (0)
#8	97	No	0 (0)

## Discussion

In mammalian species, the vitrification of germ cells is an essential tool in various basic biology and clinical areas. Since genome-editing technology has become available even for domestic species, the vitrification of embryos at the PN stage will be more important for the efficient production of genome-edited domestic animals. In this study, we succeeded in the production of pig offspring derived from vitrified PN embryos.

Vitrification is simpler and quicker than the cryopreservation technique called the conventional freezing method [31]. Successful vitrification depends on several factors. One of the factors affecting the survival and developmental ability of vitrified oocytes/embryos is how the device or protocol is used for vitrification. An increased volume of vitrification solution interferes with the survival of vitrified-warmed embryos, because a large volume decreases the cooling rate [31]. The use of only a small volume of vitrification solution in the container is thus a key to achieving vitrification at a high success rate. Many devices and methods have been developed to decrease the total volume of vitrification solution [14]. We previously succeeded in a more efficient production of offspring derived from vitrified-warmed mouse oocytes [17, 32], PN mouse embryos [21], rat oocytes [33], and PN rat embryos [34] using the Cryotop method. In matured pig oocytes, the Cryotop method yielded the highest survivability compared to other vitrification methods [11, 35]. These results of our present study strongly suggested that the Cryotop is one of the most powerful and superior devices for vitrification, even in PN pig embryos.

Although we previously succeeded in the generation of offspring derived from unfertilized mouse oocytes vitrified with EG alone because EG showed lower toxicity than DMSO [17, 32], the developmental ability of PN pig embryos vitrified with EG alone was low. Many reports support the idea that pig oocytes are more sensitive than mouse oocytes to physiological stress such as cooling and warming [11, 36, 37]. It was suggested that many lipids exist in the cytoplasm of pig oocytes, which negatively affect developmental ability after thawing [38].

Nagashima *et al*. [39] demonstrated that the physical removal of lipids (delipation) dramatically improved the developmental ability of cryopreserved pig embryos. A latter study confirmed that the uneven distribution of lipids by centrifugation can improve the developmental ability of cryopreserved embryos [22]. In the present study, we also used centrifugation for the uneven distribution of PN pig embryos, but the developmental ability was still low. These results suggest that additional improvements such as the use of a CPA are required for successful vitrification.

In this study, we used COOH-PLL as a CPA for the first time for the vitrification of embryos in a domestic species, and our findings demonstrated that the vitrification with EG and COOH-PLL dramatically improved the embryonic developmental ability *in vitro* and succeeded in production of offspring. Our previous study showed that the vitrification of mouse oocytes with EG and COOH-PLL was successful in mouse oocytes [20]. It has been reported that higher concentration of CPA such as EG increases toxicity of cells after cryopreservation but up to 20% (w/v) of COOH-PLL did not decease the survivability of frozen-thawed cells [19]. In addition, recent studies demonstrated that COOH-PLL inhibited of ice crystallization and recrystallization during freezing and thawing with anti-freezing protein-like activities *in vitro* [40, 41]. Although detailed mechanism of how COOH-PLL works in the vitrification of mammalian oocytes and embryos is still unclear, inhibition of growth of crystallization and recrystallization during vitrification seems to be a benefit using COOH-PLL.

On the other hand, developmental ability of mouse oocytes vitrified with COOH-PLL alone was very low [20] because permeability of COOH-PLL was low in mouse oocytes [20]. Higher concentration of EG had detrimental effect on survivability of the cells [19]. Therefore, the concentration of EG can be decreased by using COOH-PLL as a CPA, results in the improvement of developmental ability of vitrified embryos. We also demonstrated improvement of embryo vitrification at PN [21], 2-cell, morulae and blastocysts in the mouse (Kawasaki *et al*., unpublished data). We reported that FITC-tagged COOH-PLL entered to mouse oocyte cytoplasm to some extent [20]. In the present study however, FITC-tagged was observed in PN embryos of mouse embryos to some extent but not pig embryos (Fig 2). It is very difficult to explain the reason why there is a species-dependent differences in the permeability of COOH-PLL. However, in our previous study, vitrification solution composed of 15% EG and 15% COOH-PLL was effective for vitrification of mouse PN embryos [21]. In case of pig PN embryos, 30% EG and 20% COOH-PLL was required for successful vitrification. These differences seem to be from species-dependent permeability. In addition, one of the positive effects of COOH-PLL for vitrifcaiton of pig embryos may be to protect the membrane from cryoinjury by binding to the membrane as other non-permeable CPAs do [42, 43] and/or by inhibition of crystallization and recrystallization as described above.

Taken together, our present findings show for the first time that COOH-PLL is effective as a CPA for embryo vitrification, even in the pig. Our findings will also contribute to the improvement of oocyte/embryo vitrification in other domestic species.
